# Simple metal under tensile stress: layer-dependent herringbone reconstruction of thin potassium films on graphite

**DOI:** 10.1038/srep10165

**Published:** 2015-05-11

**Authors:** Feng Yin, Sampo Kulju, Pekka Koskinen, Jaakko Akola, Richard E. Palmer

**Affiliations:** 1 Nanoscale Physics Research Laboratory, School of Physics and Astronomy, University of Birmingham, Edgbaston, Birmingham, B15 2TT, UK; 2 School of Physics and Information Technology, Shaanxi Normal University, Xi’an 710062, PR China; 3 Department of Physics, Tampere University of Technology, P.O. Box 692, FI-33101 Tampere, Finland; 4 COMP Centre of Excellence, Department of Applied Physics, Aalto University, FI-00076 Aalto, Finland; 5 Nanoscience Center, Department of Physics, P.O. Box 35, FI-40014 University of Jyvaskyla, Finland

## Abstract

While understanding the properties of materials under stress is fundamentally important, designing experiments to probe the effects of large tensile stress is difficult. Here tensile stress is created in thin films of potassium (up to 4 atomic layers) by epitaxial growth on a rigid support, graphite. We find that this “simple” metal shows a long-range, periodic “herringbone” reconstruction, observed in 2- and 3- (but not 1- and 4-) layer films by low-temperature scanning tunneling microscopy (STM). Such a pattern has never been observed in a simple metal. Density functional theory (DFT)simulations indicate that the reconstruction consists of self-aligned stripes of enhanced atom density formed to relieve the tensile strain. At the same time marked layer-dependent charging effects lead to substantial variation in the apparent STM layer heights.

The invention of the diamond anvil cell in 1958 provided the means to study the behavior of materials under high pressure^1^,^2^. Since then high pressure measurements have revealed many interesting properties (and new phases) of various materials^3^,^4^,^5^,^6^,^7^,^8^,^9^,^10^,^11^,^12^,^13^. However, measuring these properties is problematic when the required stress is tensile rather than compressive and the sample dimensions lie in the nanoscale regime. One approach to create tensile stress is to grow thin films of the target material heteroepitaxially upon a well-defined solid surface, such that the adhesion with the surface constrains the lattice constant of the deposited layers.

One pertinent material system of particular interest is potassium (K) on graphite. This system is important both because of the ubiquity of the free electron (“jellium”) paradigm in solid state physics^14^,^15^,^16^,^17^ and because of its role as a ‘classic’ model system for adsorption^18^,^19^,^20^,^21^,^22^,^23^,^24^,^25^,^26^,^27^, also relevant to the doping of graphene^28^,^29^,^30^,^31^,^32^. In addition, K-graphite intercalation compounds are superconductors^31^,^33^. Previous investigations on complete monolayers of K on graphite at temperatures below 90 K have shown a close-packed (2 × 2) structure^18^,^19^,^20^,^21^,^22^,^23^,^25^,^26^,^27^. The large difference of 8.4% between lattice dimensions^26^,^34^ plus the symmetry mismatch between the hexagonal graphite surface and bulk bcc structure of the K crystal enables the generation of tensile strains in thin films of this “simple”, prototypical “jellium”-like metal. In this work, we employ low-temperature scanning tunneling microscopy (LT-STM) imaging and density-functional simulations to reveal and interpret the emergence of bright stripe reconstructions in 2- and 3- layer K films. The reconstructions, together with charge states and apparent layer heights, depend critically on the number of layers and display a powerful manifestation of the underlying tensile stress.

## Results

**Figure 1:**
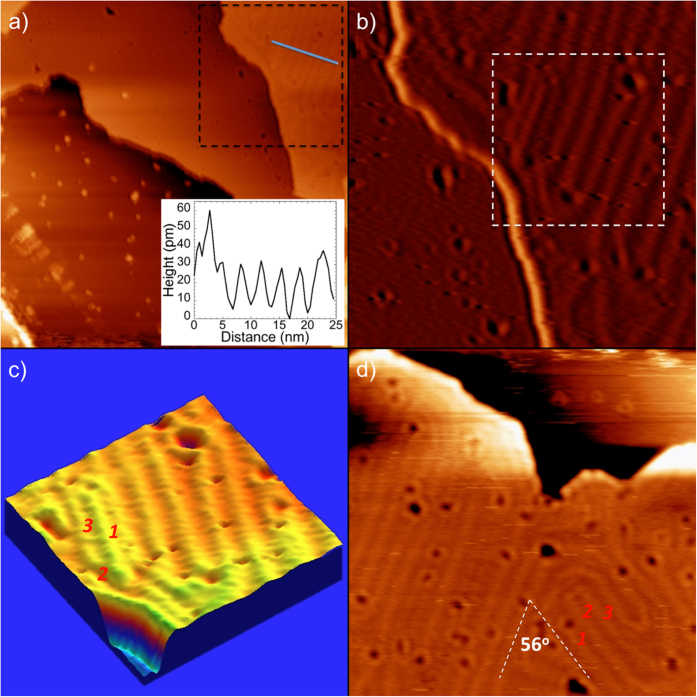
(**a**) Constant current STM (at −2.0 V, 15 pA) image (133 nm × 133 nm) of multilayer K film on graphite; inset, the line profile of the region indicated by the line on the figure. (**b**) The zoomed-in STM topographic image (55.1 nm × 55.1 nm) taken from the black dashed box in (**a**). It was obtained by applying a derivative filter to the image. (**c**) Three-dimensional visualization of the STM image (27.3 nm × 27.3 nm) taken from the white dashed box in panel b. Numbers denote different domains. (**d**) Constant current STM (at −2.0 V, 15 pA) image (66.4 nm × 66.4 nm) of one of the second K layer islands.

Figure 1a shows the topography of a K/graphite sample, which exhibits K coverage up to two layers, as well as some small K islands distributed on otherwise bare regions of the graphite surface. The step edges are rough, indicating a high density of kink and corner sites. Some “pockmarks”, each surrounded by a bright ring, can be seen both in the first and second K layers. A notable difference between the layers is that the two-layer film shows a long-range, periodic, bright stripe pattern, while the one-layer film does not. In a zoomed-in image (enhanced by applying a digital derivative filter), the pattern on the second K layer becomes especially visible (Fig. 1b). The height of the bright lines is about 30 pm above the K layer and the distance between two parallel lines is about 3.5 nm (inset of Fig. 1a). The 3D visualization, Fig. 1c, shows the stripe pattern even more clearly, and also reveals slight undulations along each stripe, which looks rather like a spinal cord. The stripe pattern resembles the herringbone reconstruction observed on the (111) surface of Au^35^,^36^, and similar structures in several other fcc metal heteroepitaxial systems and the nitrogen-covered (110) surface of Cr are assigned to misfit dislocation networks^37^,^38^,^39^,^40^. Such a pattern has never been observed in a “free electron” metal.

The concept of a traditional fcc herringbone reconstruction is problematic in the case of a simple metal. The traditional herringbone always presents alternating hcp and fcc domains (lateral change in stacking)^35^,^36^. Consider by contrast the 3D visualization of the 2-layer film in Fig. 1c, where the labels 1, 2, and 3 represent three adjacent domains. If we assume, for the sake of argument, that domain 1 is an “fcc” domain, then its immediate neighbours (domains 2 and 3) ought to be “hcp” domains (we note that formally it is not possible to distinguish fcc and hcp for only two layers, and our discussion refers to different stacking sites for the upper layer). However, domains 2 and 3 are also both immediate neighbors - and by the same logic cannot be of the same kind! The same phenomenon can be seen in domains 1, 2 and 3 in Fig. 1d. Moreover, the bright lines of the striped pattern run along two different orientations (Fig. 1d). The angle between the orientations is about 56^o^, which is smaller than the angle of the herringbone reconstruction of an fcc metal (60^o^). These observations suggest that the stripe pattern on K film is not a traditional herringbone reconstruction.

**Figure 2:**
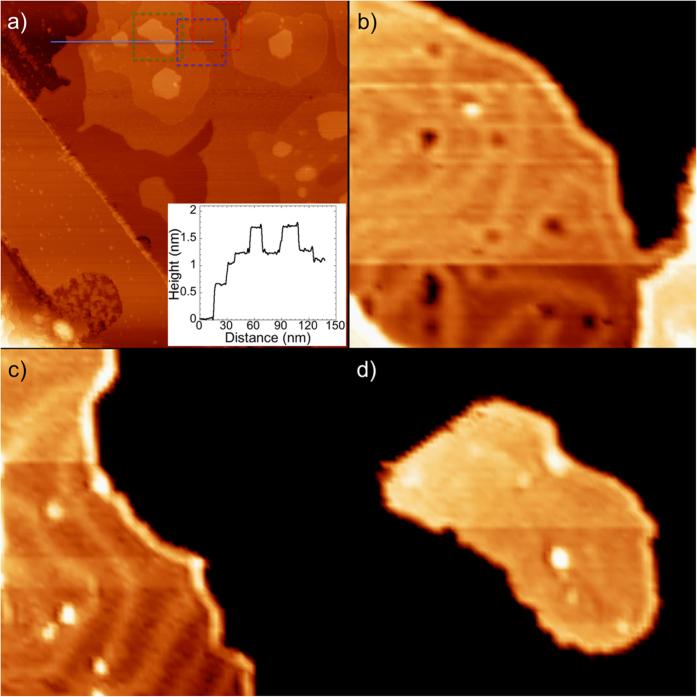
(**a**) Constant current STM (at −2.0 V, 15 pA) image (215 nm × 215 nm) of K multilayer film on graphite. Inset, the line profile of the region indicated by the line on the figure. (**b**) The STM topographic image (40 nm × 40 nm) of the 2nd layer of the K film taken of the area within the red dashed box in (**a**). (**c**) The STM topographic image (40 nm × 40 nm) of the 3rd layer of the K film taken of the area within the blue dashed box in (**a**). (**d**) The STM topographic image (40 nm × 40 nm) of the 4th layer of the K film taken of the area within the green dashed box in (**a**).

The behavior of the K film depends drastically on the number of atomic layers, as demonstrated by another area of the same sample that shows 3- and 4-layer K islands (Fig. 2a). Here the third and fourth layers are almost atomically flat, which indicates good layer-by-layer growth, though again with rough step edges. A zoom-in image shows that the periodic bright stripe pattern appears on the 3-layer film (Fig. 2c) similar to the 2-layer film (Fig. 2b), but not on the 4-layer film (Fig. 2d). The atomic STM image shows that the 4-layer film is a typical close-packed structure^41^ (for more detail, see Supplementary Information). We note also that the apparent heights of the K layers in STM vary considerably. As shown in the line profile (inset to Fig. 2a) the first layer is ~0.6 nm high, the second layer has a smaller apparent height of ~0.4 nm, and the third layer has the smallest height, ~0.2 nm. The height of the fourth K layer increases again to ~0.5 nm. The statistical evaluation of the step heights confirms this strong variation in the apparent heights of the K layers (for more detail, see Supplementary Information). These height differences and the appearance and then disappearance of the stripe pattern with increasing film thickness imply that the atomic and electronic structures of the 1-, 2-, 3-, and 4-layer K films must be mutually different.

**Figure 3:**
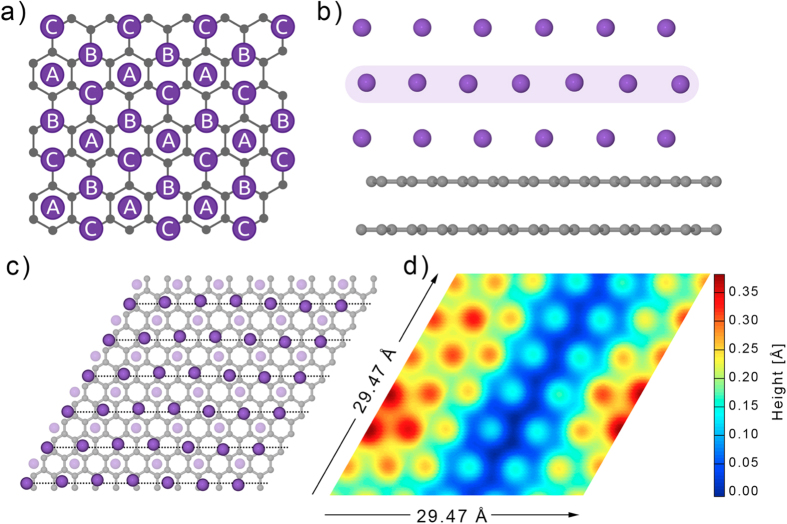
Atomistic model of a 3-layer K film on HOPG in a hexagonal simulation cell of 29.47 × 29.47 Å^2^ (**a**) Schematic image of K layers on graphite with the possible adsorption sites A, B, and C. (**b**) Side view of a simulated slab system, where the second K layer has an extra row of atoms. (**c**) Top view of the same slab with the second K layer highlighted by a darker shade; notice the bending of the lines (with respect to the dashed straight lines). (**d**) Simulated STM image of the same geometry for an isosurface approximately 5 Å above the surface (bias voltage −2.0 eV).

Previous experimental^18^,^19^,^20^,^21^,^22^ and theoretical^23^,^26^,^27^ investigations of monolayer K on graphite at temperatures below 90 K show that the close-packed (2 × 2) phase of the complete first layer has the same symmetry as a (111) plane of an fcc lattice. This means that the K atoms occupy sites A in the schematic view of K/graphite shown in Fig. 3a. The distance between two A sites is 4.92 Å and the angle between two close-packed directions is 60^o^. If the second K layer is commensurate with the first, the second layer K atoms should occupy hollow sites of the first layer (B sites in Fig. 3a). But what is the effect of the tensile stress in the system? How will the atoms arrange in multilayer films and what is the connection to bulk K? These are the questions that we now address, seeking to interpret the appearance of the novel herringbone structure.

Our assumption is that the formation of misfit dislocations is induced by the incorporation of additional atoms into the second K layer, to increase the surface atom density. The nearest neighbour distance between the sites in the (2 × 2) lattice on graphite (4.92 Å) is considerably larger than the metallic diameter of the K atom (4.54 Å)^42^; it may be that the openness of this first layer not only provokes the incorporation of additional K atoms to increase the lateral density, creating the observed dislocations, but also allows the second layer to sink, accounting for the reduced inter-planar spacing normal to the structure (STM height changes from ~0.6 nm to ~0.4 nm as discussed above). The measured 56^o^ angle between the two stripe orientations further indicates that the second K layer is still near (but not quite) hexagonal, this is similar to the unreconstructed bcc (110) lattice. A K atom in the third layer has two options in occupying the hollow sites of the second layer (sites C and A in Fig. 3a), corresponding to fcc and hcp packing. At this thickness the misfit strain between the third layer and the bulk K is (based on the experimental results) still strong enough to induce the striped pattern. Since the striped pattern cannot be observed on the fourth layer, and the STM height of the fourth K layer recovers back to ~0.5 nm, we can infer that the strain in the fourth K layer is relaxed and the K atoms are accommodated in an fcc (or hcp) crystal structure.

## Discussion

In order to provide insight into the interplay between different atomic arrangements, density functional (DF) simulations of K films on graphite were performed with the CP2K/Quickstep method (for more detail, see Supplementary Information)^43^,^44^,^45^,^46^. Several geometries were tested for 2-4 layer K films on a hexagonal graphite support. For reference, close-packed films based on the (2 × 2) monolayer construction on graphite were simulated for each film thickness: for a 3-layer film we included both stackings, ABA (hcp) and ABC (fcc), according to the labeling of Fig. 3a. Various “defect” structures were tested, including single atom vacancies and interstitials, in-plane stacking changes (fcc/hcp), and extra rows of atoms embedded in the layers. The summary of results for different structures of a 3-layer film is given in Table 1 and shows, for example, that the energy difference between ABA (hcp) and ABC (fcc) stacking is negligible. Furthermore, the defect formation energies show that interstitial defects become less favourable as the K film becomes thicker (for more detail, see Supplementary Information), because translational periodicity (via second neighbor interactions) becomes more important in thicker films. This explains why the herringbone pattern disappears in the experiments for 4-layer films that follow the fcc/hcp stackings and still reflect the underlying (2 × 2) lattice spacing on graphite.

The most interesting, and also the lowest-energy, “defect” structure emerging from the calculations of 3-layer films corresponds to the ideal ABA configuration with an extra row of atoms (in C sites) placed in the second layer (Fig. 3b–d). This structure (system V in Table 1) is only 6 meV/atom less favourable energetically than the ideal ABC and ABA configurations (systems I and II). Laterally, this structure shows a slight variation in the atomic positions (darker magenta atoms in Fig. 3c). In particular, it shows STM height variations of up to 0.4 Å in different locations on the film surface, in agreement with the experimental STM results. Although the defect row is in the second layer, it still causes a visible undulation in the electron density of the top layer. The spacing of 2.55 nm between the periodic “stripes” is smaller than in the experiments, but this is merely because of the size of the simulation box. Interestingly, similar extra row defects placed within the top layer (systems III and IV) are slightly higher in energy, indicating that it is energetically favourable for a 3-layer system to bury defects below the surface. Also, lateral changes in the stacking from A to B domains – the traditional herringbone reconstruction – creates free sites in between domains for an additional row of atoms (system VI) and makes the cohesive energy the least favourable (−19 meV/atom) and the lateral height variation (~2 Å) an order of magnitude too large to fit the STM measurements. Note that while the fcc and hcp systems I and II match the chosen hexagonal unit cell (Fig. 3) perfectly, the energies for defect structures could possibly be decreased by increasing the cell dimensions. In other words, the close-packed layers are preferred by the choice of our simulation cell and periodic boundary conditions. Our theoretical results indicate that the energy cost for forming in-plane atomic line defects in K layers is very small (if any), and that the resulting simulated and experimental STM height variations are similar in magnitude.

To account for the observed 3.5 nm separation of stripes and 56^o^ angles between stripe orientations, more extended lateral models are required, and we have demonstrated one such case in the Supplementary Information (figure S5 in Supplementary Information). This model demonstrates for a 2-layer system how the herringbone pattern appears *spontaneously* as we include the atomic concentration of bcc (110) in the upper layer (more atoms than in the close-packed (2 × 2) layer). The structure is only 5 meV/atom higher in total energy than the close-packed 2 ML case, and it agrees well with the experiments by showing a stripe pattern with STM height variations around 0.5 Å (atomic variation 0.3 Å) and ~3.5 nm separation of the stripes. A closer inspection of the atomic structure reveals that the “stripe” regions correspond to linearly arranged line defects in the deformed top layer, while the intermediate regions adjust to the underlying hexagonal layer.

**Figure 4:**
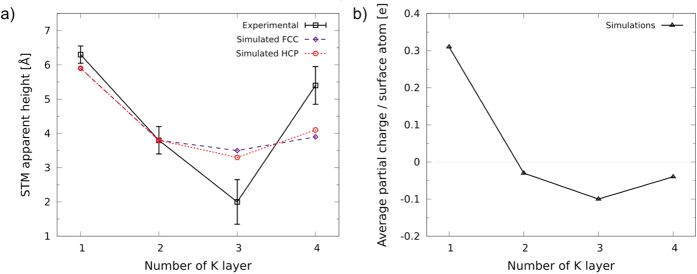
(**a**) Experimental and simulated STM layer heights for the topmost K layer as a function of film thickness. (**b**) Average partial charge of the surface atoms for different number of layers. Partial charge is presented in units of elementary charge.

The apparent heights of the layers of different thicknesses in STM were simulated with the CP2K/Quickstep program using a surface bias voltage of −2 V and an *s*-orbital for the tip. The reference value of the STM density isosurface was calibrated to the experimental STM height of the second K layer. As seen in Fig. 4a, the apparent height values obtained in the simulation show the same trend as the experimental ones. The contrast with bare graphite is highly visible because the apparent height of the first layer is more than 2 Å larger than any other K layer. The apparent height of the third layer is significantly lower than the second and fourth. The electronic effect in the STM is much stronger than the pure geometrical effect; geometrical heights are all between 3.5-4.0 Å for 2-4 ML (for more detail, see Supplementary Information). Thus the K monolayer separation from the graphite surface is drastically overestimated by STM due to (positive) layer charging effects.

Bader charge analysis for the layers reveals charging of the surface atoms, and this is visible from the partial charge per surface atom in Fig. 4b. There is a significant charge transfer from the lowermost K layer to graphite, while the upper layers display slight charge accumulation. The average partial charge per surface atom shows a notable correlation with the simulated layer heights, which is physically plausible. Moreover, the electrostatic field of the tip is likely to enhance the STM height oscillation even further with respect to the simulations where such effect is not included^26^.

In conclusion, epitaxial growth of a simple metal (potassium) on a “rigid” graphite substrate generates a tensile stress which profoundly influences the atomic structure. The “soft” simple metal film adopts the (111) surface of a face-centered cubic (fcc) crystal (K-K distance 4.92 Å, graphite (2 × 2) coverage) rather than the denser (110) surface of the bulk bcc structure in 1- and 4- layer films. The stress is relieved by the formation of a stacking fault in the second layer, leading to the appearance of a long-range periodic stripe pattern on the second and third potassium layers in the STM images. DFT simulations indicate that this feature is due to additional K atoms which result in linearly arranged line defects, *i.e.* stripes. The layer dependent charging effect also induces considerable height differences in the STM. The phenomena observed provide a fundamental new insight into the behaviour of simple metals under extreme conditions (of tensile stress) as well as the behaviour of “jellium” surfaces close to instability and suggest that similarly rich structural and electronic behaviour may also be observed in other epitaxial simple metal films.

## Methods

### Sample preparation and STM manipulation/image

To prepare the K/graphite specimens, highly oriented pyrolytic graphite (HOPG) samples were cleaved with tape just before loading into an ultra high vacuum (UHV) chamber (base pressure 1.3 × 10^−10^ mbar), where they were annealed for 30 minutes at 450 ^o^C. Such graphite surfaces display clean, atomically flat terraces in STM imaging. After annealing the graphite was cooling down to 90 K by liquid nitrogen and potassium was evaporated onto the surface by dosing for 30 seconds with a commercial getter source (SAES Getters). Then, the samples were transferred in UHV into the LT-STM chamber with a base pressure of 1.1 × 10^−11^ mbar. The STM imaging was conducted at 4.4 K by an Omicron LT-STM with a mechanically sheared Pt/Ir (90/10) tip and the STM images were analysed by WSxM software^47^.

### Density functional simulations

Density functional (DF) simulations of K overlayers on HOPG were performed using the CP2K method ( http://www.cp2k.org)^45^,which uses both localized Gaussian functions and plane waves as basis sets. For the Gaussian-based (localized) expansion of the Kohn-Sham orbitals we used a library of contracted molecularly optimized valence double-zeta plus polarization (m-DZVP) basis sets^46^, and for the complementary plane wave basis set we used a cutoff energy of 400 Ry (used for electron density only). The exchange-correlation energy functional employed the PBE parametrization of the generalized gradient-corrected approximation^43^, and the valence electron–ion interaction was based on norm-conserving and separable pseudopotentials^44^. We considered the valence configurations 3s^2^3p^6^4s^1^ for K and 2s^2^2p^2^ for C. Periodic boundary conditions were used in all directions, with a single ***k***-point (***k ***= 0) in the Brillouin zone. The selected sampling of the reciprocal ***k***-space is appropriate for the purpose of this work, where the lateral size of the extended simulation box is 29.5 Å with 288 C atoms per graphene layer. In practice, this means that the initial Brillouin zone of graphite is extensively folded, and the effects of varying the *k*-point mesh have been demonstrated, for example, in Ref. 25 giving us confidence that the chosen approach is valid. The vertical dimension of the cell was selected to be large enough to isolate the slab replica. The K films were geometrically optimized, while the HOPG support was kept fixed. Due to electrostatic binding, the dispersion forces between K films and HOPG make a very small contribution and there is no need for additional van der Waals functional; PBE captures the essential physical phenomena and is a valid functional for the given purpose. Simulated STM images were computed with a surface bias voltage of −2 V (same as in the measurements) and an *s*-orbital for the tip. The image was plotted as a density isosurface with a value taken approximately 5 Å above the surface. This mimicks the constant current mode of an STM tip (density fixed, tip height varied).

## Author Contributions

F. Y. and R. E. P. initiated this work, S.K., P. K., and J. A. performed the modelling and simulation, F. Y. built experimental set and performed the experiments, S.K., P. K., and J. A. wrote the simulation parts of the manuscript, F. Y. and R. E. P. wrote the other parts of the manuscript. All authors reviewed the manuscript.

## Additional Information

**How to cite this article**: Yin, F. *et al*. Simple metal under tensile stress: layer-dependent herringbone reconstruction of thin potassium films on graphite. *Sci. Rep.* **5**, 10165; doi: 10.1038/srep10165 (2015).

## Supplementary Material

Supplementary Information

## Figures and Tables

**Table 1:** Comparison of calculated film geometries for a 3-layer K film on graphite in a hexagonal cell 29.47 Å square.

System	**Geometry**	**E**_**C**_ **[eV/at.]**	**∆E [meV/at.]**	**d**_**┴**_**(K-C) [Å]**	**r**^**top**^**(K-K) [Å]**	**∆h [Å]**
I	ABA	0.917	—	3.02	4.91	—
II	ABC	0.916	−1	3.04	4.91	—
III	AB(A + C line)	0.903	−14	2.97	4.61	0.45
IV	AB(C + A line)	0.904	−13	2.96	4.57	0.31
V	A(B + C line)A	0.911	−6	2.97	4.91 (4.58)	0.21 (0.12)
VI	AB(A/C/A)	0.902	−15	3.02	4.64	1.96

The geometries are labelled according to the stacking sequence starting from the bottom layer (A). The line defects initially placed in either the A or C positions (Fig. 3a). The values in parentheses correspond to the defect layer. The cohesive energies E_C_ and its changes with ∆E are shown per K atom. Structural parameters shown: d_┴_(K-C), separation between the first K layer and graphite; r^top^(K-K), average lateral K-K bond distance within the most top layer; and ∆h, height variation corrugation within in the top most K layer.
