# Supplementary material for: Simple metal under tensile stress: layer-dependent herringbone reconstruction of thin potassium films on graphite
Source: Sci Rep. 2015 May 11;5:10065. doi: 10.1038/srep10165 (PMC4426671; doi:10.1038/srep10165)
Supplement: Supplementary Information [file srep10165-s1.pdf]

# Supplementary information: Simple metal under tensile stress: layer-dependent herringbone reconstruction of thin potassium films on graphite

*Feng Yin*[1, 2], *Sampo Kulju*[3, 4], *Pekka Koskinen*[5], *Jaakko Akola*[3, 4], *and Richard E Palmer*[1*]

[1]*Nanoscale Physics Research Laboratory, School of Physics and Astronomy, University of Birmingham, Edgbaston, Birmingham, B15 2TT, UK*

[2]*School of Physics and Information Technology, Shaanxi Normal University, Xi'an 710062, PR China*

[3]*Department of Physics, Tampere University of Technology, P.O. Box 692, FI-33101 Tampere, Finland*

[4]*COMP Centre of Excellence, Department of Applied Physics, Aalto University, FI-00076 Aalto, Finland*

[5]*Nanoscience Center, Department of Physics, P.O. Box 35, FI-40014 University of Jyvaskyla, Finland*

## ATOMIC RESOLUTION IMAGE AND THE ANGLE BETWEEN TWO DISCOMMENSURATION

Figure. S1 shows an atomic resolution STM image from a 4th–layer K surface with the same STM tip. From this image, we can see that the experimental image agrees very well with the hexagonal lattice. It indicates that the fourth layer of K displays is a close-packed surfaceand the distortion of STM imaging is negligible in this case. Moreover, in order to obtain the accurate value of the angle between the two orientations of discommensuration lines, we measure 12 angles on the 2nd-layer terrace in forward- (figureS2a) and backward- (figureS4b) scanning STM images (same area as figure 1d in main manuscript). We can see that the angles are distributed from 55.3 degrees to 57.6 degrees. Comparing the two images, we can see that the two images agree very well. So we can conclude that the angle between the orientations is ~56 degrees.

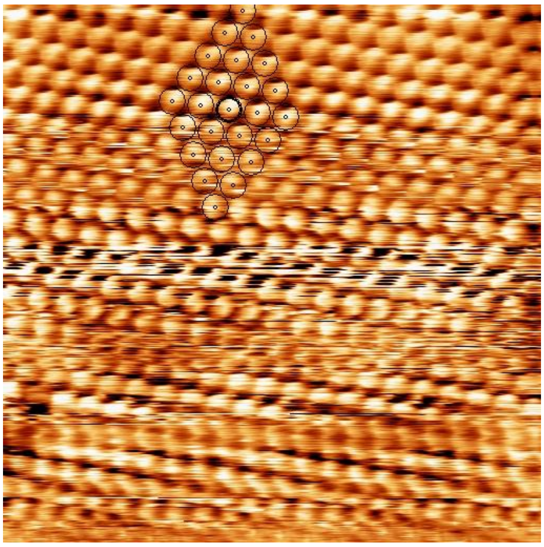

**Figure S1.** Atomic resolution STM (at -1.6 V, 100 pA) image (9 nm × 9 nm) of a fourth layer terrace. The agreement between the hexagonal lattice and the experimental image indicates that the fourth layer of K displays a close-packed surface.

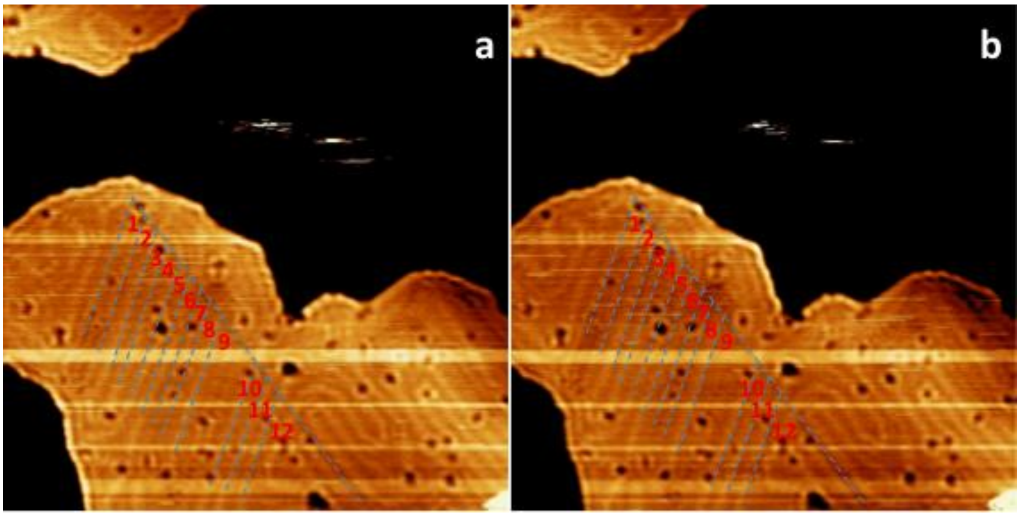


**Figure S2.**Constant current STM (at -2.0 V, 15 pA) images (100 nm × 100 nm) of one of the second K layer islands, (a) Forward scanning. (b) Backward scanning. The value of the 12 angles is 1: 55.8°, 2: 56.5 °, 3: 57.6 °, 4: 55.5 °, 5: 56.1 °, 6: 56.5 °, 7: 56.0 °, 8: 56.5 °, 9: 56.6 °, 10: 55.3 °, 11: 55.9 °, 12: 56.4 °

## STATISTICS OF THE HEIGHT OF POTASSIUM LAYERS

The apparent height distribution in the red box demarcated on STM image of K/graphite (figure S3a) was counted and displayed in figure S3b. In this figure, we can see five peaks correspondingto the apparent height of the blank graphite surface and the four K films. In order to estimate the apparent height of the four K films, we simulated the five peaks with the Lorentz distribution. The Lorentz fitting peaks agree with the experimental results very well(figure S3b). The parameters of the five Lorentz fitting peaks and the apparent heights of the four K layers are listed in Table S1. Here the FWHM of the Lorentz fitting peaks is used

as the error.

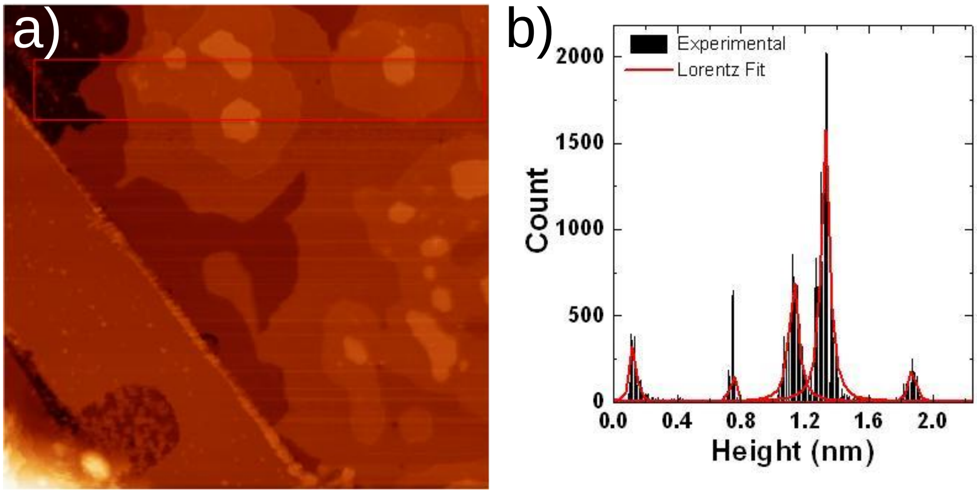


**Figure S3.** (a) Constant current STM (at -2.0 V, 15 pA) image (215 nm × 215 nm) of K multilayer film on graphite. (b) Apparent height distribution in the area ismarked by the red box in (a) and Lorentz fitting peaks.

**Table S1.**The parameters of the five Lorentz fitting peaks and the apparent heights of four K layers

| Peak | Centre (Å) | Width (Å) | Height of layer (Å) |
|---|---|---|---|
| 1 | 1.2 | 0.40 | - |
| 2(1$^{st}$ layer) | 7.5 | 0.10 | 6.3±0.25 |
| 3(2$^{nd}$ layer) | 1.13 | 0.70 | 3.8±0.40 |
| 4(3$^{rd}$ layer) | 1.33 | 0.60 | 2.0±0.65 |
| 5(4$^{th}$ layer) | 1.87 | 0.50 | 5.4±0.55 |

## BRIGHT STRIPE PATTERN IN SMALL 3$^{RD}$-LAYER TERRACE

Figure S4 shows the STM image of multilayer K film and the zoom-in images from 3rd-layer (figure S4b) and 4th-layer (figure S4c) terraces. We can see the bright stripe pattern in the small 3rd-layer terrace (~30nm).However, such a pattern cannot be seen in the 4th–layer terrace, which has a similar size (~30nm). It indicates that the formation of such bright stripe patternsis independent of the size of the terrace.

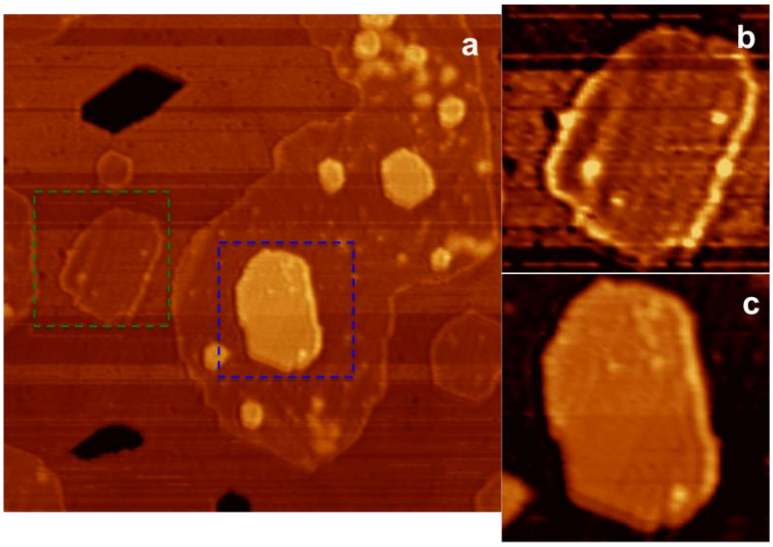


**Figure S4.**(a) Constant current STM (at -2.5 V, 10 pA) image (150 nm × 150 nm) of multilayer K film on graphite. (b) A 3rd-layer terrace (39.8 nm × 39.8 nm), zoomed-in from the black dashed box in (a). (c) A 4th-layer terrace (39.8 nm × 39.8 nm), zoomed-in from the blue dashed box in (a).

## THEORETICAL STM IMAGE OF THE LATERALLY EXTENDED 2-LAYER SYSTEM

At this point it is worthy remarking that the almost hexagonal (110) plane of the unreconstructed bcc bulk structure of K has similar symmetry to the close-packed hexagonal layers. However, the distances between two nearest neighbours in the bcc lattice are 5.32 Å along the [100] direction (8.1% difference with respect to the (2 × 2) K/graphite monolayer) and 4.61 Å along the [111] direction (-6.3% difference). The angle between [100] and [111] directions is 54.75°. An interesting idea is the possible formation of bcc (110) layers (bulk potassium) on top of hexagonal layers. Correspondingly, we have simulated a laterally enlarged 2-layer K film on HOPG with an orthorhombic unit cell (39.30 Å × 59.55 Å). The cell dimensions were selected to match both the upper bcc K (110) layer and the underlying hexagonal (2 × 2) K structure. The bcc (110) layer comprised 5 atoms more than the hexagonal layer in this particular simulation cell. Small distortion of the initial coordinates resulted in a spontaneous relaxation into another structure with a lower total energy (only 5 meV/atom higher than that of the hexagonal AB structure). This structure agrees well with the experiments by showing a stripe pattern with STM height variations around 0.5 Å (atomic variation 0.3 Å) and ~3.5 nm separation of the stripes (figure S5). A closer inspection of the atomic structure reveals that the "stripe" regions correspond to line defects in the deformed top layer, while the intermediate regions adjust to the underlying hexagonal layer (figure S5b-c). A similar optimized structure (and energy) was achieved by rearranging atoms in the

initial bcc (110) layer in linear stripes (see below).

We wish to emphasize that the emergence of the stripe-like pattern occurs spontaneously in this model and is a direct consequence of introducing the density of bulk K in the top layer. The choice of a unit cell to fit both the bcc (110) surface atoms and hexagonal layer (beneath) enables the emergence of the stripe pattern. However, the theoretical description obtained is not perfect as the diagonal stripes are not continuous, and we believe that this is due to the periodic boundary conditions, as the experiment indicates that the angle between the stripes deviates from 60°. In other words, the orientation of the stripe pattern does not coincide with the hexagonal symmetry, and indeed we can observe a mismatch in the locations of maxima in the periodically repeated images (figure S5). We assume that larger atomic models (unit cells) may well resolve this problem, and that the connection between the herringbone pattern and linearly arranged line defects in the top K layer would appear even more visibly. However, as noted in figure 1(main text), some undulation along the stripes seems to be apparent in the experimental STM images too. Finally, our calculations for 4-layer systems indicate that the single atom defects, as well as additional “lines”, become energetically less favourable, explaining the disappearance of stripes in the STM images.

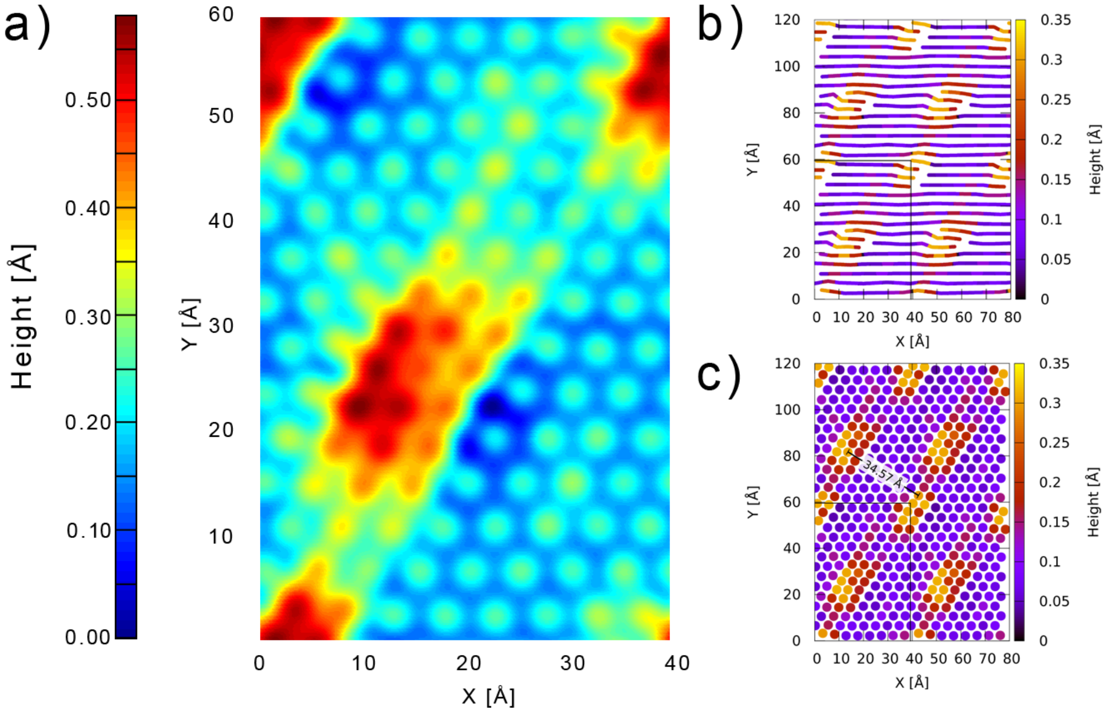


**Figure S5.** 2-layer K film on HOPG in a large rectangular unit cell of 39.30 Å × 59.55 Å. (a) Simulated STM image for an isosurface approximately 5 Å above the surface (bias voltage -2.0 eV). (b) A 2×2 replicated cell to illustrate the structure of line defects. Colors correspond to height variations. (c) Topology of the slab, 2×2 replicated cell.

## SIMULATED LAYER HEIGHTS IN STM IMAGES

The STM layer heights for different number of monolayers were simulated using a surface

bias voltage of -2 V and an $s$-orbital for the tip (main text, figure 4). The reference value of the STM density isosurface, however, was calibrated to the experimental STM height of the second layer. Here an isovalue was chosen so as to obtain the STM height for the second layer as closely as possible to the experimental one. As seen from the layer heights thus obtained, the simulated values have the same trend as the experimental ones (Table S2). The contrast with plain graphite is visible as the first layer is more than 2 Å thicker than any other K layer, and the second and the fourth layers are thicker than the third one. The STM effect is more enhanced than the geometrical effect, where the variations of layer height are between 3.5-4.0 Å for 2-4 ML. The K monolayer separation from the graphite surface is drastically overestimated by STM due to charging effects.

Bader charge analysis for the multilayer systems revealed charging of the surface atoms, and this is visible from the partial charge per surface atom (Table S2). The partial charges are presented in units of elementary charge. The average partial charge per surface atom shows correlation with the simulated layer heights.

**Table S2.** Simulated and experimental STM heights, average partial charges per surface atom (negative means more electrons at surface), and geometrical layer heights, all for different number of monolayers. For 3 and 4 monolayers the stacking is indicated in parentheses.

| Geometry | Experimental STM height [Å] | Simulated STM height [Å] | Layer height from geometry [Å] | Average partial charge / surface atom [e] |
|---|---|---|---|---|
| Graphite | - | - | - | 0.00 |
| 1 ML | 6.3±0.25 | 5.9 | 3.1 | 0.31 |
| 2 ML | 3.8±0.40 | 3.8 | 3.8 | -0.03 |
| 3 ML (ABC) | 2.0±0.65 | 3.5 | 3.6 | -0.10 |
| 3 ML (ABA) | | 3.3 | 3.5 | -0.10 |
| 4 ML (ABCA) | 5.4±0.55 | 3.9 | 3.9 | -0.04 |
| 4 ML (ABAB) | | 4.1 | 4.0 | -0.04 |

## FORMATION AND COHESIVE ENERGIES

The formation energy $E_{form}$ was calculated for an interstitial site on the surface with the equation

$$E_{form} = E_{inters} - E_{ideal} - E_{singleK} \ ,$$

where$E_{inters}$ is the total energy of the system with the defect, $E_{ideal}$ is the energy of ideal system and $E_{singleK}$ is the energy of single potassium atom in the same simulation cell. Similarly, we compute the vacancy formation energy as

$$E_{form} = E_{ideal} - E_{vacancy} - E_{singleK} ,$$

where$E_{vacancy}$ is the total energy of the system with a single vacancy on the surface.

Cohesive energy $E_{coh}$ was calculated with the equation,

$$E_{coh} = \frac{-(E_{tot} - E_{graph} - N \cdot E_{singleK})}{N},$$

where$E_{tot}$ is the total energy of the system, $E_{graph}$ is the energy of the graphite slab in same calculation cell, $N$ is the number of potassium atoms and $E_{singleK}$ is the energy of single potassium atom in the same calculation cell.

**Table S3.**Formation and cohesive energies for systems with an atom in the interstitial site and with a vacancy in the surface layer.

| Geometry | Stacking | $E_{coh}$ / K [eV] | $E_{form}$[eV] |
|---|---|---|---|
| 2 ML + interstitial | AB | 0.908 | -0.604 |
| 3 ML + interstitial | ABA | 0.913 | -0.490 |
| 3 ML + interstitial | ABC | 0.913 | -0.567 |
| 4 ML + interstitial | ABAB | 0.900 | -0.418 |
| | | | |
| 2 ML with vacancy | AB | 0.913 | -0.817 |
| 3 ML with vacancy | ABA | 0.915 | -1.155 |
| 3 ML with vacancy | ABC | 0.915 | -1.090 |
| 4 ML with vacancy | ABAB | 0.904 | -0.888 |

## FURTHER INFORMATION OF ATOMIC MODELS

In addition to defect rows, the stack change structure was tested in the same hexagonal unit cell of lateral size 29.47 Å for both two and three layer systems with two graphene layers (see main text, Table 1, system VI). For example, for the bilayer system the second layer was

changed from B stacking to C and lines in the stacking change region were placed higher from the surface plane. After geometry optimization the structure had one line in the third layer and second layer had same number of atoms than ideal (2×2) layer (stack change still visible). The starting and optimized geometry are presented in figure S6. The resulting totalenergies of such stack change systems were higher (unfavourable) than for the other systems with the defects (lines). Furthermore, this construction is not a plausible reason for the herringbone pattern because the line in the third layer is more than 3 Å higher than the second layer, which is not comparable to experimental data of the STM heights. The same effect was observed for the optimized 3-layer system with stack change, although the height change had reduced to 2 Å.

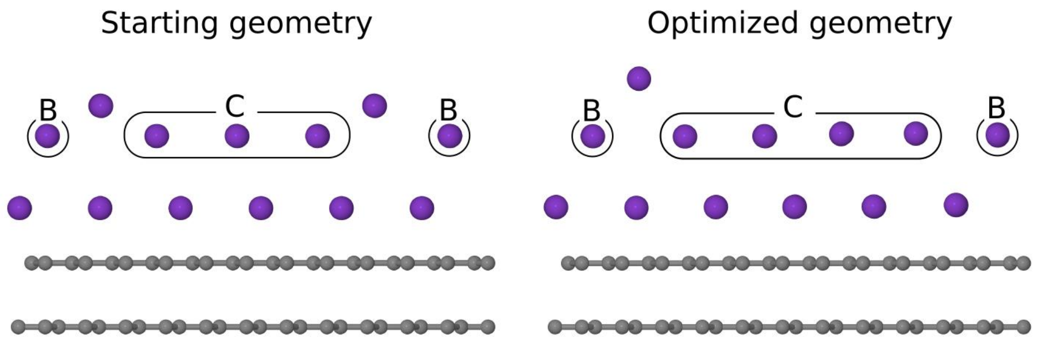


**Figure S6**. Starting and optimized geometry of a K bilayer in the hexagonal calculation cell of 29.47 Å (*a* side, hexagonal symmetry) with stack change in top layer. Stacking of the atom rows are presented in the figure with corresponding letters. After optimization the atom density in second layer is same than in first "ideal" layer, but the stack change is still visible.

A laterally larger unit cell was used in order to be able to simulate a potassium bcc (110) layer on a (2×2) commensurate potassium layer and support. At first, the bcc (110) layer and (2×2) layers were placed artificially close to each other, and the system relaxed to a structure resembling the herringbone pattern. The effect was lost and the bcc (110) layer stayed intact (figure S7) when the distance between the layers in the starting structure was increased to a more natural level (no compression). The resulting cohesive energy per K atom was noticeably less favourable (lower) than that for the defected structure.

Next idea was to consider the lateral interplay between different lattices; hexagonal (2×2) vs. bcc (110). Atoms were moved in the topmost bcc (110) layer to B-stacking sites (figure S8) to form (2×2) stripes and bcc (110) region between them. After geometry relaxation, this structure shows approx. 30 pm height variation and a stripe pattern with 3.5 nm separation, as forthe results presented in figure S5. The high regions (lines) are formed due to atomic line defects, and their origin is in the concentration of atoms, i.e. there are extra atoms in the bcc (110) layer compared to the hexagonal (2×2) layer. For this unit cell size, the difference is 5

potassium atoms in a layer compared to that of a pure (2×2) structure, as shown previously for a similar system in figure S5.

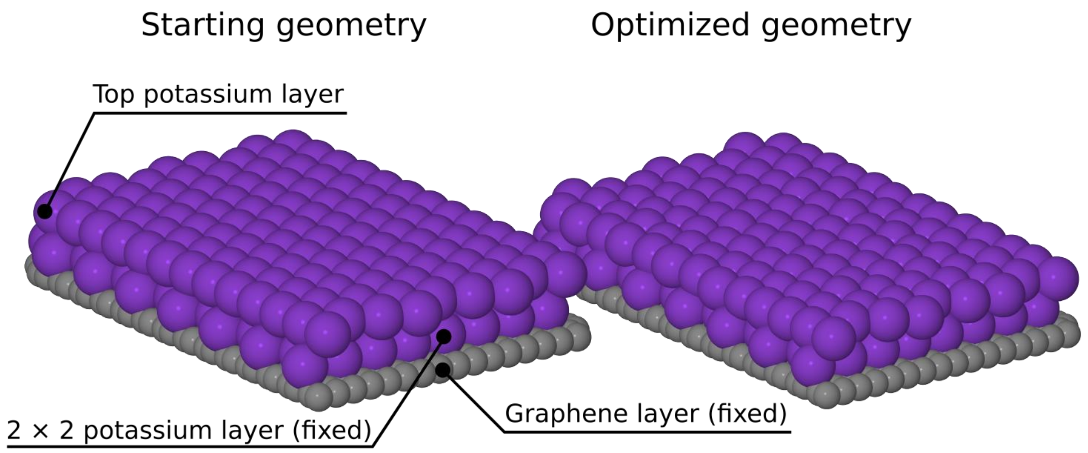


**Figure S7.**Starting and optimized geometry of the large unit cell comprising a (2×2) potassium layer on graphene and a topmost layer of bcc (110). As can be seen, the top layer keeps it bcc (110) structure upon geometry optimization. The corresponding total energy is higher than for the structure with line defects.

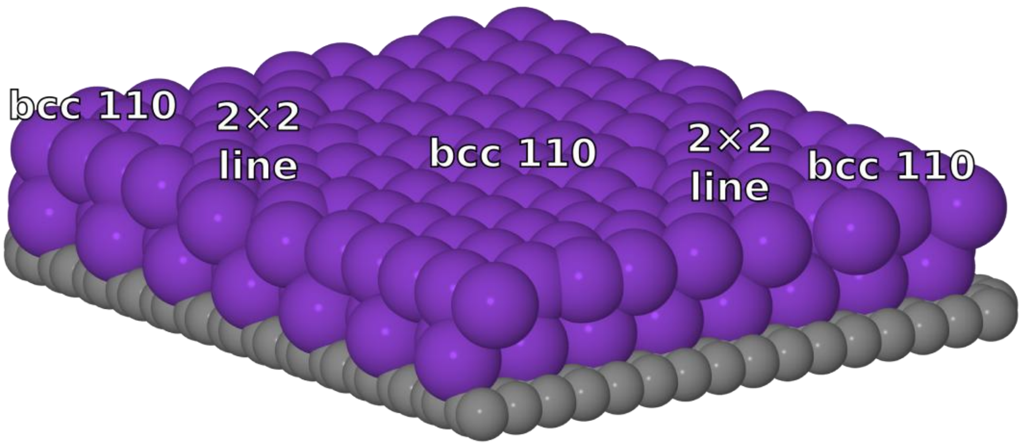


**Figure S8.** Starting geometry for large system with displaced atoms ((2×2) stripes). Lateral size of the orthogonal unit cell is 39.30 Å × 59.55 Å.

## CONSTRUCTION OF GEOMETRIES

The (2×2) potassium layer was based on a previously optimized structure in a smaller hexagonal cell. This was expanded periodically to form a larger slab. The bcc (110) layer was cut out from the bulk structure and put on the (2×2) layer after a suitable rotation along the surface normal. The unit cell size was selected to fulfill periodic boundary conditions for all the layers (graphite, (2×2) potassium and bcc (110) potassium). With these boundaries the cell size is 39.30 Å × 59.55 Å and the total number of atoms is 1125. The number of K atoms is

229, where 112 atoms are in the (2×2) layer and 117 in the bcc (110) layer. To reduce the simulation load, a single layer of graphene was used as a (fixed) support, and the lower (2×2) potassium layer was kept fixed also.
